# Double Trouble: The Burden of Child-rearing and Working on Maternal Mortality

**DOI:** 10.1007/s13524-020-00868-6

**Published:** 2020-04-08

**Authors:** Tabea Bucher-Koenen, Helmut Farbmacher, Raphael Guber, Johan Vikström

**Affiliations:** 1 ZEW - Leibniz Centre for European Economic Research, University of Mannheim and MEA, L7,1, D-68161 Mannheim, Germany; 2 Munich Center for the Economics of Aging (MEA), Max Planck Society, Amalienstr. 33, D-80799 Munich, Germany; 3 Institute for Evaluation of Labour Market and Education Policy (IFAU), P.O. Box 513, SE-751 Uppsala, Sweden

**Keywords:** Mortality, Maternal health, Fertility, Twins

## Abstract

We document increased old-age mortality rates among Swedish mothers of twins compared with mothers of singletons, using administrative data on mortality for 1990–2010. We argue that twins are an unplanned shock to fertility in the cohorts of older women considered. Deaths due to lung cancer, chronic obstructive pulmonary disease, and heart attacks—all of which are associated with stress during the life course—are significantly increased. Stratifying the sample by education and pension income shows the highest increase in mortality rates among highly educated mothers and those with above-median pension income. These results are consistent with the existence of a double burden on mothers’ health resulting from simultaneously child-rearing and working.

**Electronic supplementary material:**

The online version of this article (10.1007/s13524-020-00868-6) contains supplementary material, which is available to authorized users.

## Introduction

In times of demographic change and aging populations, policy-makers in many countries are concerned about the development of the work force. In order to buffer the systematic aging and potential shrinking of the work force, policies that encourage an increase in female labor supply are often on the agenda. At the same time, family policies aiming at an increase in fertility rates are discussed (Jaumotte 2004). However, for many mothers (and arguably fathers), simultaneously raising children and pursuing a career is challenging because both pursuits are time-consuming. Moreover, balancing family life and careers may affect parental health through stressors occurring from the double burden of working and caring for children. We investigate this (potential) double burden and its effect on maternal health later in life. When designing public policies aimed at increased fertility rates and/or increased female labor supply, any negative health consequences of family and work have to be taken into account. In the United States and many other developed countries, work-family conflict and its potential negative effects are a public concern (see, e.g., Williams and Boushey 2010).

Our analysis is based on administrative birth and death registries from Sweden. To study the effect of childbearing and labor force activity on maternal health, an ideal set-up would provide exogenous variation in both labor market activity and childbearing. In our study, we use twin births as an unplanned fertility shock. Initially, we compare health outcomes of mothers who had twins at first birth (hereafter referred to as *twin mothers*) with those of mothers of singletons at first birth (*nontwin mothers*). The Swedish data allow us to follow mothers over a long period, and to our knowledge, we are the first to study the effects of twins on mothers’ old-age mortality. We find that twin mothers have a 3.8 percentage point (13%) higher all-cause mortality risk compared with mothers of singletons. This finding provides some initial evidence on the relationship among family, work, and health. However, although twinning is an exogenous shock,^1^ it can affect old-age health via multiple pathways. The twin mothers in our data have, on average, 0.68 more children five years after the first birth, but child spacing also changes: the spacing between the first and the last child is 3.8 years for twin mothers and 7.4 years for nontwin mothers. Twinning may also affect health risks during pregnancy and delivery as well as marital stability. Moreover, if women adjust their labor market choices depending on childbearing (Lundborg et al. 2017), this might reduce any negative health impacts from the fertility shock due to twinning. Therefore, we examine the reduced-form effect and do not use twin births as an instrumental variable for the number of children.

To understand potential mechanisms and to study a double-burden effect of family and work, we pursue two strategies.^2^ First, we stratify the sample along educational attainment and pension income, both of which are strongly related to labor force attachment. Level of education is an *ex ante* predictor of higher labor market activity, and pension income is a proxy for *ex post* realized labor market activity. Thus, if the double burden of working and caring for children is important, we expect to see larger health effects of a fertility shock (twin births) for women with high education and/or high pension income because these women more often combine working and child-rearing.^3^

The second strategy to understand the mechanism behind the increased mortality of twin mothers is to analyze specific stress-related diseases. The underlying hypothesis is that the double burden of working and caring for children at the same time creates stress, which may particularly affect stress-related mortality. Here, one challenge is the measurement of lifetime stress. We analyze two specific groups of medical diagnoses related to stress during life: cardiovascular diseases (heart attacks and strokes) and smoking-related diseases (lung cancer and chronic obstructive pulmonary disease (COPD) (Brotman et al. 2007; Kouvonen et al. 2005). The literature shows that strong predictors of cardiovascular diseases are known to be elevated by chronic stress, which results from caregiving, for example.^4^ Stress from work-family conflicts is also related to smoking behavior (Hurtado et al. 2016; Nelson et al. 2012), which in turn is strongly correlated with lung cancer and COPD. If these stress-related causes of death are affected, we take this as evidence of increased stress due to the double burden.

We acknowledge that level of education may interact with child-rearing in ways other than through the stress caused by the double burden of family and work. For instance, highly educated mothers receive higher incomes, which in turn may allow them to better cope with the increased stress. Higher education is also associated with jobs that offer more flexible working hours and more control over work content, which may help to alleviate conflicts between children and jobs. In this study, we do not attempt to disentangle these different mechanisms behind the relationships among level of education, labor supply, child-rearing, and maternal health. Unfortunately, we lack data on important factors, such as work content and control over work time, because we do not know which jobs mothers in our sample held in the past. However, most of these alternative mechanisms—such as income and work conditions—suggest that highly educated mothers are better able to compensate for the twin burden. Nevertheless, we see that highly educated mothers are more affected by twinning, suggesting that the double burden of family and work outweighs these other mechanisms; we take this as evidence of a double-burden effect of family and work. In robustness analyses, we also use additional data to examine some of these alternative mechanisms.^5^

Given that we compare twin and nontwin mothers, all women in our sample have at least one child. Thus, we are able to study the effect of fertility only at the intensive margin—that is, comparing having a singleton versus twins at first birth. A comparison at the extensive margin (i.e., having any children versus none) would be at least as interesting as the intensive margin comparison: by definition, women without children do not experience the double burden of children and work. However, it is hard to find exogenous variation on the extensive margin effect. In fact, in a lone exception, Lundborg et al. (2017) used the success of *in vitro* fertilization (IVF) treatments to obtain exogenous variation at the extensive margin. Interestingly, they found that the effects of fertility on earnings are larger at the extensive margin than at the intensive margin. Given this evidence, it seems reasonable to extrapolate that there also are important double-burden effects at the extensive margin.

Our study is linked to the literature on the interactions among fertility, working life, and maternal health. Kahn et al. (1964) were among the first to note that a conflict between work and nonwork roles are a major source of stress. A large literature has researched the work-family conflict empirically. However, many early studies were based on small or highly selective samples (Greenhaus and Beutell 1985). Studies based on large, representative, or administrative data include Weatherall et al. (1994), Martikainen (1995), and Waldron et al. (1998). More specifically, related to our study, Nelson et al. (2012) found a strong relationship between work-family conflicts and smoking behavior in a sample of English long-term care workers. Similarly, Väänänen et al. (2005) documented a negative association between conflicts due to paid and domestic work and health among men and women. Using retrospective data from the Health and Retirement Study, Sabbath et al. (2015) categorized mothers along their past marriage, fertility, and working histories, concluding that working single mothers experience the highest mortality rates in old age. Van Hedel et al. (2016) and Berkman et al. (2015) found that single working motherhood is associated with higher likelihood of stress-related heart diseases. Although suggestive of a double-burden effect, these studies and other related studies were not able to address self-selection of women into specific work-family profiles based on their health and other unobserved related factors. By using twins at first birth as a fertility shock, we try to overcome this limitation.

A growing literature has examined the relationship between family life and female careers. Several studies found that childbearing has negative effects on female labor force participation, earnings, and wages (Angrist and Evans 1998; Lundborg et al. 2017) and that male and female earnings diverge after the birth of the first child (Angelov et al. 2016; Bertrand et al. 2010; Kleven et al. 2019a, b). Other studies examined how public policies, such as parental leave policies, affect labor force participation and childbearing (Del Boca 2002; Lalive and Zweimüller 2009; Schönberg and Ludsteck 2014) or sickness absence from work (Guertzgen and Hank 2018). We study a related aspect of family life and female careers: the double burden of simultaneously working and caring for children.

Two closely related studies are Cáceres-Delpiano and Simonsen (2012) and Kruk and Reinhold (2014). Using multiple births as an instrumental variable, Cáceres-Delpiano and Simonsen (2012) found that a higher number of children implies worse health for U.S. mothers aged 20–45. Based on data from the Survey of Health, Ageing and Retirement in Europe, Kruk and Reinhold (2014) showed that an increase in the number of children has a negative impact on mental health of older women but no effect on older men. Kruk and Reinhold used twin births and sibling sex composition as instruments for the number of children. We add to this literature by focusing on heterogeneity in the long-term effects of fertility on health. We are the first to study effects of fertility on (cause-specific) old-age mortality. In addition, unlike Cáceres-Delpiano and Simonsen (2012) and Kruk and Reinhold (2014), we rely on administrative rather than survey and census data.

## Data

We use the Swedish multigeneration register, which links all individuals to their biological mother (and father), even if they do not live in the same household or have died. The register contains parental information for persons born in 1932 or later, including information on year and month of birth (for further details about the register, see Ekbom 2011). Twins are identified as being born to the same mother in the same year and month as another sibling.

From the registry, we identify 404,286 mothers aged 55–65, alive, and resident in Sweden in 1990. Of those mothers, 2,684 (0.66%) had twins at first birth.^6^ We follow the mothers for 20 years, from 1991 to 2010. From the National Causes of Death Register, we know whether they died and, if so, the cause of death. We focus on two groups of diseases that may be related to stress during life: cardiovascular diseases and smoking-related diseases. For the former, we study heart attacks and strokes; for the latter, lung cancer and COPD. Here, we follow the strategy by Evans and Moore (2012) to classify the diagnoses into specific disease categories.^7^

Among all mothers born between 1925 and 1935, 8.6% were not observed in 1990 because they died (75%) or moved abroad (25%) before 1990. In Table A1 of the online appendix, we investigate whether twin and nontwin mothers differ in the probability to be included in our study sample (column 1) or in the probability of dying before 1990 (column 2); we find no significant differences. Thus, although the sample as a whole may suffer from survival/migration bias, our results are unlikely to be biased because twin and nontwin mothers are affected symmetrically.

We have a set of socioeconomic characteristics from population registers. Table 1 describes our variables for the full sample and stratified by mothers’ educational attainment. Education is defined in three categories: primary, secondary, and tertiary schooling. *Primary schooling* includes elementary schooling (*Folkskola*), which comprised seven years for most of the cohorts considered here, and junior secondary schooling (*Realskola*). The designation of *secondary schooling* means that mothers had some education beyond the primary level but no tertiary education. This level also includes vocational training at the upper secondary level. *Tertiary schooling* indicates that mothers experienced some higher education: that is, the attainment of some college or of a university education or a PhD.

**Table 1:** Descriptive statistics by mothers’ education

	Full Sample	Primary Schooling	Secondary Schooling	Tertiary Schooling
Age (1990)	60.03	60.34	59.74	59.24
	(3.16)	(3.13)	(3.16)	(3.06)
Age at First Birth	24.56	23.92	24.81	27.13
	(4.67)	(4.55)	(4.62)	(4.43)
Number of Children	2.40	2.45	2.32	2.36
	(1.21)	(1.30)	(1.11)	(1.00)
Twins at First Birth (in %)	0.66	0.64	0.66	0.82
	(0.08)	(0.08)	(0.08)	(0.09)
Same-Sex Twins at First Birth (in %)	0.44	0.42	0.44	0.55
	(0.07)	(0.06)	(0.07)	(0.07)
Employed (1990 in %)	66.00	57.08	74.82	88.74
	(0.47)	(0.50)	(0.43)	(0.32)
Died Between 1991 and 2010 (in %)	28.72	31.81	26.16	19.48
	(0.45)	(0.47)	(0.44)	(0.40)
Died From Lung Cancer or COPD (in %)	4.46	5.02	4.15	2.40
	(0.21)	(0.22)	(0.20)	(0.15)
Died From Heart Attack or Stroke (in %)	13.61	15.70	11.85	7.53
	(0.34)	(0.36)	(0.32)	(0.26)
*N*	404,286	237,558	120,340	46,388
%	100.00	58.76	29.77	11.47
Pension Income at Age 72 in 100 SEK	817	676	871	1,253
	(471)	(429)	(410)	(468)
Pension Income Above Median (in %)	50.00	39.15	53.48	80.96
	(0.50)	(0.48)	(0.49)	(0.39)
*N*	209,325	109,650	69,102	30,573

*Notes:* Standard deviations are shown in parentheses. Primary schooling is defined as education levels 1 and 2; upper secondary schooling, as levels 3 and 4; and tertiary schooling, as levels 5, 6, and 7.

On average, the mothers were 60 years old in 1990, had their first child at age 24.5, and have 2.4 children. The majority of the mothers completed primary education (59%), about 30% completed secondary education, and roughly 11% earned a tertiary degree. The age at first birth is, on average, three years higher in the highly educated group compared with the lower-educated group, and the average number of children is about the same.

Overall, about 66% of the women aged 55–65 are still active on the labor market (positive labor income) in 1990. However, the fraction of working women varies considerably by education. Whereas 89% of the women with a tertiary degree receive labor income, only 57% of mothers with primary schooling receive income from work at those ages. About 29% of the women in our sample died between 1991 and 2010, with large variation by education. About one-third of the lower-educated mothers died in the 20-year time window we consider, compared with only 26% (19%) among mothers with medium (high) levels of education. We see similar patterns for the different causes of death.

From the population registers, we also obtain information on pension income at age 72. Pension income is the best available proxy for lifetime labor force participation in our data set.^8^^,^^9^ Pension income at age 72 follows the expected pattern; the mean pension income of mothers with tertiary schooling is more than 125,000 SEK and approximately 85% higher than the pension income of mothers with primary schooling or less.^10^

## Empirical Strategy

To investigate differences between twin and nontwin mothers, we specify the following linear regression model:1where *y*_*i*_ is the outcome variable, and  is an indicator equal to 1 if mother *i* gave birth to twins at first birth. Depending on the specification, the outcome variables are indicators equal to 1 if the mother died from any cause between 1991 and 2010, from a heart attack or stroke, or from lung cancer or COPD between 1991 and 2010. The control variables, **x**_***i***_, include dummy variables for seven education levels, dummy variables for mothers’ birth cohorts, and a quadratic polynomial in age at first birth.^11^^,^^12^

One worry when comparing twin mothers with nontwin mothers is that twinning may not be entirely random. For instance, Bhalotra and Clarke (2016) documented that twin mothers are positively selected with respect to health and health behaviors. However, Farbmacher et al. (2018) showed that this selection issue is primarily attributable to dizygotic (fraternal) twins. Dizygotic twins become more likely with increasing age of the mother (Fauser et al. 2005; Reddy et al. 2005) and the use of IVF treatments (Thurin et al. 2004), creating a correlation between twin births and maternal health. Monozygotic twins, on the other hand, are considered to be truly random (MacGillivray et al. 1988; Tong and Short 1998).

**Fig. 1:**
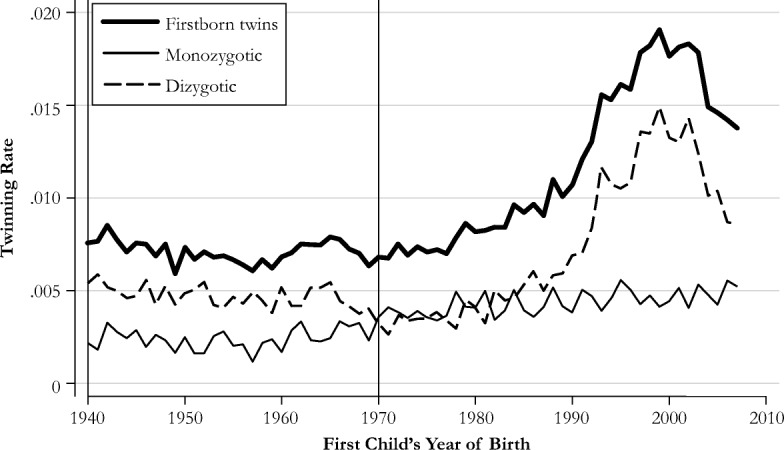
Twin rate in Sweden for firstborn children. Statistics are based on the Swedish register data. To compute the monozygotic and dizygotic twinning rates, we apply Weinberg’s (1901) rule. The vertical lines indicate the period in which more than 99% of the mothers in our sample gave birth to their first child.

We have several strategies for addressing these issues. First, as mentioned earlier, we control for age at first birth in all our analyses. Second, to investigate a possible selection bias stemming from dizygotic twins, we compare estimates between mothers of all twins and only same-sex twins (while also controlling for age at first birth). Because monozygotic twins necessarily have the same sex, their share must be higher among same-sex twins (Black et al. 2007; Figlio et al. 2014). Third, IVF is less of a concern in our data given that more than 99% of the mothers gave birth to their first child between 1940 and 1970, when IVF treatment was not yet available. This is illustrated by Fig. 1, which shows the twin rates in Sweden across the first child’s year of birth. The share of twins remained fairly constant between 1940 and 1980 but increased strongly thereafter. Although the steady but mild rise in the twin rate after 1980 could be attributed to delayed childbearing, the steep increase in the twin rate since 1990 mainly follows the availability of IVF. The vertical lines in the graph indicate the cohorts included in our analysis by birth year of the first child(ren).

Twinning affects the number of children the mother has over a specific period and after completed fertility. However, whereas many previous studies used the birth of twins as an instrumental variable for fertility, we study the so-called reduced-form effects of twinning. That is, we estimate differences between twin and nontwin mothers, thereby capturing all mechanisms through which twinning affects mortality. Along with the main mechanism—the increase in the number of children—other effects may exist. First, twin pregnancies and delivery, on average, pose a greater health risk to the mother than singleton births, which may translate into higher old-age mortality rates (Buhling et al. 2003; Rauh-Hain et al. 2009). Second, because twins are extremely closely spaced, having twins may influence the spacing of further children, which in turn might have a direct effect on mother’s health. Third, twinning may affect the probability of divorce, which may act as an additional channel of stress. Fourth, mothers may adjust their labor market choices if they have twins (Lundborg et al. 2017), perhaps by decreased labor force participation, which in turn may have health effects. We therefore focus on the overall effect of twinning and interpret any effects as a general effect of child-rearing. As a background, we also show how twinning affects completed fertility and birth spacing.

To analyze the double-burden effect, we study the effect of twinning for subsamples defined by level of education and pension income. Education is an important predictor of labor force participation, working hours, and earnings because highly educated mothers are more prone to pursue careers. Therefore, we expect that given the same unplanned fertility shock, highly educated mothers are more likely to experience a double burden of working life and child-rearing. Thus, a finding of larger health effects of twinning for highly educated mothers would be evidence of a double-burden effect. Note that we can use education for stratifying the analysis because most of the mothers in our sample completed their education before giving birth to their first child; the education level is less influenced by fertility than is labor force participation itself.^13^

We use twinning as a fertility shock and assume that highly educated mothers experience more stress because they are more likely to work. However, level of education is also correlated with other factors that may influence the effect of twinning on maternal health. First, the Grossman (1972) model predicts that highly educated individuals are better at using medical care goods and services and are therefore more able to mitigate negative health effects. Second, highly educated mothers receive higher incomes, which may allow them to better cope with the increased stress due to twinning. Third, higher education is also associated with jobs that offer more flexible working hours and more control over the work content, which may also help alleviate conflicts between child-rearing and work. Fourth, because of educational homogamy, fathers’ involvement may differ between higher- and lower-educated mothers. Fifth, because higher education induces more specialized jobs, highly educated women might move farther from their families and have different networks. However, most of these alternative mechanisms indicate that highly educated mothers should be less affected by twinning. In other words, a finding that highly educated mothers are more affected by twinning would suggest that the double burden of family and work outweighs these other mechanisms. Thus, even if we are not able to completely disentangle the different mechanisms behind the relationships among level of education, labor supply, child-rearing, and maternal health, we can provide some evidence on the double burden of family and work.

Although higher education holds the *ex ante* potential only for greater labor force attachment, pension income is a proxy for lifetime income and is thus an *ex post* realization of the former. Pension income in Sweden is independent of the partners’ income. Our hypothesis is that mothers with a higher pension income are more likely to experience a double-burden effect. We therefore stratify the sample at the median pension income, with an income greater than the median implying more labor market attachment. Pension income is a direct result of labor market participation, which could be reduced because of childbirth. Thus, pension income is an endogenous outcome. However, pension income is also the best available proxy for lifetime labor force participation in our data set, and we believe that splitting the data set based on it reveals valuable insights. One caveat of the split by pension income is that a specific level of pension income can result from working many hours at a medium or low wage as well as working few hours at a high wage. A high number of hours worked is usually what creates the conflict with child-rearing and thus the double burden. However, if women with low and high pension income do not differ in the number of hours worked but only in their average income, we should see no differences in the effect of having twins by pension income. Additionally, twin births could have a direct effect on survival and retirement until age 72, which may create selective sorting. Table A1 in the online appendix (columns 4–5) shows no differences between twin and nontwin mothers with respect to whether the pension income at age 72 is missing or the amount of pension income at age 72.

Our results are derived for the cohorts of mothers born between 1925 and 1935 who had their first children between 1940 and 1970. To get an idea how these cohorts differ from younger cohorts in terms of mother’s age at first birth, the number of children, and birth spacing, we plot the main differences by mothers’ birth cohorts in the online appendix (Figs. A1–A4). For the cohorts born between 1925 and 1935, age at first birth declined from around 25 years to about 24 years, remaining there until around age 24 for the birth cohort until the late 1940s and increasing continuously thereafter (Fig. A1, online appendix). The number of children per mother fluctuated between just more than 2.4 and 2.2 children per mother for all cohorts, with the highest average number of children among women born in the early 1930s and the lowest for women born around 1945 (Fig. A2, online appendix). Birth spacing between the first and the second as well as the first and the last child declined continuously for mothers until the cohorts born around 1940. It increased slightly for mothers born until 1950 but continued to decrease thereafter (Figs. A3 and A4, online appendix).

## Results

### Twins and Old-Age Mortality

**Fig. 2:**
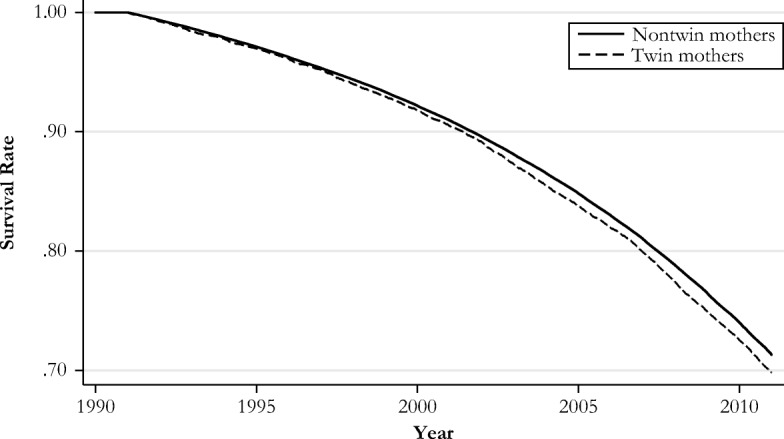
Survival rates for mothers with and mothers without twins at first birth

We now turn to our main analysis. Figure 2 presents Kaplan-Meier survival curves for mothers with and without twins. A clear gap in the survival probabilities emerges between the two groups over the 20-year period. Twin mothers are dying at a higher rate compared with their peers who only had one child at first birth. The gap becomes larger around the year 2002: that is, when the women in our sample are on average 72 years old.

Panel A of Table 2 contains estimates for the effect of having twins at first birth based on the regression model from Eq. (1). Having twins at first birth increased the probability of dying by 3.7 percentage points over a 20-year period. Relative to a baseline probability of dying of 28.7%, twin mothers had a 13% higher mortality rate than nontwin mothers. This pattern holds for specific causes of death (see columns 2 and 3). Twin mothers were 1.3 percentage points (20%) more likely to die of lung cancer or COPD compared with other mothers. Their likelihood of dying from a heart attack or stroke was 1.8 percentage points (13%) higher during the period of observation.

**Table 2:** Main results: Coefficients from linear probability models controlling for education, cohort dummy variables, and a quadratic polynomial in age at first birth

	(1) Died Between 1991 and 2010	(2) Lung Cancer/COPD	(3) Heart Attack/Stroke	(4) Femur Fracture
A. Twins	0.037**	0.013**	0.018**	0.005
	(0.009)	(0.004)	(0.007)	(0.004)
B. Same-Sex Twins	0.038**	0.013*	0.020*	0.005
	(0.011)	(0.005)	(0.008)	(0.005)
Unconditional Mean	0.287	0.044	0.136	0.047
Number of Observations	404,286	404,286	404,286	404,286

*Notes:* Each coefficient–standard error pair comes from a separate regression. In panel A, the reference group is mothers with a singleton first birth. In panel B, the reference group is mothers with singletons or opposite-sex twins at first birth. Robust standard errors are shown in parentheses.

*p < .05; **p < .01

As robustness checks, we estimate a placebo regression on femur fractures, which are unlikely to be affected by stress during working life; they are usually caused by severe accidents or, for older adults, by falls. The placebo outcome is equal to 1 if the mother had a femur fracture in the period 1991–2005. As shown in panel A of Table 2 (column 4), the prevalence of femur fracture is indeed not significantly higher among twin mothers. Panel B shows the estimate when only same-sex twins are used as treatment, excluding potentially nonrandom opposite-sex (dizygotic) twins from the treated group. The estimate is very similar to that in panel A. Both robustness checks suggest that our results are not driven by direct effects of twinning on health or nonrandom selection into twinning.^14^

To provide background for these estimates, Tables A2 and A3 (online appendix) report statistics on the number of children and child spacing for twin and nontwin mothers. Table A2 shows that twins at first birth lead to a substantially larger number of children in the short run as well as in the long run. Five years after the first birth, twin mothers have, on average, 0.68 more children than nontwin mothers; 15 years after the first birth, this difference is 0.59 children. As expected, twinning also affects child spacing. Table A3 shows that the average time between the first and the second child is approximately 4 years for nontwin mothers (0 by construction for twin mothers). Twinning also affects the spacing between the first and the last child, which is 3.8 years for twin mothers and 7.4 years for other mothers. Our twinning estimates thus capture the combined effects of the increased number of children, the reduced spacing, and potentially other effects that we discussed earlier.

### Results by Educational Level and Pension Income

To investigate the interaction of fertility and labor market attachment, we split the sample by education and pension income. Table 3 displays our results stratified by maternal education. Panel A shows that the effect of having twins at first birth for mothers with at most primary schooling is slightly smaller than the effect estimated for the full sample (reported in Table 2). However, the probability of dying from lung cancer, COPD, or heart diseases is slightly higher among the mothers with primary schooling compared with the overall effects. For mothers with secondary schooling, we find a similar effect of twins on overall mothers’ mortality compared with the full sample (panel B), but we find no elevated levels of lung cancer, COPD, or cardiovascular diseases for these mothers. Interestingly, the largest effect sizes in absolute and relative terms are experienced by mothers within the highest education group (panel C). For twin mothers compared with nontwin mothers, all-cause mortality is increased by 8.4 percentage points (43%), and death due to lung cancer and COPD is increased by 2.2 percentage points, which corresponds to an increase of almost 100%. Death due to a heart attack or stroke is 4.1 percentage points (55%) higher.^15^ Reassuringly, we also find that effect sizes for the estimates based on all and same-sex twins are quite similar for all specifications.

**Table 3:** Results by education: Coefficients from linear probability model controlling for the subcategories of education, cohort dummy variables, and a quadratic polynomial in age at first birth

	Died Between 1991 and 2010	Lung Cancer/COPD	Heart Attack/Stroke
	(1)	(2)	(3)
A. Primary Schooling			
Twins	0.028*	0.018**	0.021*
	(0.012)	(0.006)	(0.010)
Same-sex twins	0.027^†^	0.020*	0.023^†^
	(0.015)	(0.008)	(0.012)
Unconditional mean	0.318	0.050	0.157
Number of observations	237,558	237,558	237,558
B. Secondary Schooling			
Twins	0.032*	-0.003	0.001
	(0.016)	(0.007)	(0.011)
Same-sex twins	0.028	-0.005	-0.002
	(0.020)	(0.008)	(0.014)
Unconditional mean	0.262	0.042	0.119
Number of observations	120,340	120,340	120,340
C. Tertiary Schooling			
Twins	0.084**	0.022*	0.041*
	(0.023)	(0.011)	(0.017)
Same-sex twins	0.099**	0.020	0.055**
	(0.028)	(0.013)	(0.021)
Unconditional mean	0.195	0.024	0.075
Number of observations	46,388	46,388	46,388

*Notes:* Each coefficient-standard error pair comes from a separate regression. Robust standard errors are shown in parentheses.

^†^*p* < .10; *p < .05; **p < .01

We next split the sample at the median by pension income. As described in the Empirical Strategy section, pension income is observed for only a subsample of mothers. We first report the overall twinning effects for this reduced sample (panel A, Table 4). Here, the health effects are slightly smaller than estimates for the full sample in Table 2 but are still sizable and significant. Panels B and C of Table 4 show the effects of twins for mothers below and above the median pension income, respectively. Again, the pattern is consistent with the double-burden hypothesis. We find no significant effects of twins for mothers with below-median pension income; however, for mothers with above-median pension income, there are sizable mortality effects. This holds for both overall and cause-specific mortality. For instance, for mothers with a pension income above the median, having twins increases the probability of dying over a 20-year time period by 4.6 percentage points, or almost 40%.^16^^,^^17^

**Table 4:** Results by pension income: Coefficients from linear probability models controlling for education, cohort dummy variables, and a quadratic polynomial in age at first birth

	Died Between 1991 and 2010	Lung Cancer/COPD	Heart Attack/Stroke
	(1)	(2)	(3)
A. All, Conditional on Pension Income Observed			
Twins	0.028**	0.009*	0.013^†^
	(0.009)	(0.005)	(0.007)
Same-sex twins	0.034**	0.010^†^	0.020*
	(0.012)	(0.008)	(0.009)
Unconditional mean	0.122	0.022	0.058
Number of observations	209,325	209,325	209,325
B. Below-Median Pension Income			
Twins	0.010	0.002	0.006
	(0.012)	(0.005)	(0.009)
Same-sex twins	0.020	0.006	0.004
	(0.016)	(0.007)	(0.011)
Unconditional mean	0.124	0.020	0.061
Number of observations	104,682	104,682	104,682
C. Above-Median Pension Income			
Twins	0.046**	0.017*	0.020*
	(0.014)	(0.008)	(0.010)
Same-sex twins	0.049**	0.014	0.038**
	(0.018)	(0.009)	(0.014)
Unconditional mean	0.120	0.024	0.056
Number of observations	104,643	104,643	104,643

*Notes:* Each coefficient-standard error pair comes from a separate regression. The low-pension (high-pension) sample contains individuals with below-median (above-median) pension income at age 72, corrected for inflation. Robust standard errors are shown in parentheses.

^†^*p* < .10; *p < .05; **p < .01

As a robustness check, we also reestimate the models by using interaction terms between the twin birth indicator and education levels instead of splitting the sample. The results in columns 1–3 of Table A4 (online appendix) are very similar to those from the split-sample analyses. The same holds if we use an interaction model for the below- and above-median pension income analyses (Table A5).

We next split the sample by education and pension income into six cells; the results are presented in Table 5. Because of the lower sample size in each cell, the difference between twin and nontwin mothers is not significant in most cases, but the sign and magnitude of the coefficients are consistent with the previously presented results. In particular, the effect of twins on overall mortality is the largest for highly educated mothers with above-median pension income.

**Table 5:** Results by pension income and education: Coefficients from linear probability models controlling for finer subcategories of education, cohort dummy variables, and a quadratic polynomial in age at first birth

	(1) Primary Schooling	(2) Secondary Schooling	(3) Tertiary Schooling
A. Below-Median Pension Income			
Twins	0.000	0.034	0.001
	(0.015)	(0.024)	(0.045)
Same-sex twins	0.005	0.050	0.039
	(0.019)	(0.032)	(0.066)
Unconditional mean	0.134	0.111	0.086
Number of observations	66,719	31,143	5,820
B. Above-Median Pension Income			
Twins	0.026	0.034	0.085**
	(0.023)	(0.024)	(0.027)
Same-sex twins	0.037	0.036	0.081*
	(0.018)	(0.030)	(0.033)
Unconditional mean	0.143	0.120	0.082
Number of observations	42,931	36,959	24,753

*Notes:* The outcome variable is death between 1991 and 2010 due to all causes. Each coefficient-standard error pair comes from a separate regression. The low-pension (high-pension) sample contains individuals with below-median (above-median) pension income at age 72, corrected for inflation. Robust standard errors are shown in parentheses.

*p < .05; **p < .01

We interpret these results as evidence of a double burden of family and work. However, as we noted earlier, the level of education is also correlated with other factors that may influence the effect of twinning on maternal health. For example, higher-educated mothers receive higher incomes, which may help alleviate some stress and provide access to better health care. To test for the importance of this channel, we adjust for income in our regressions by controlling for a quadratic polynomial of log labor income in 1985.^18^ The results presented in columns 4–6 of Table A4 (online appendix) are very similar to the results from the main analyses, where we do not control for past income. This suggests that income is not the main mechanism behind our results.

## Discussion and Conclusions

In summary, we find that having twins increases all-cause mortality significantly for women older than age 55. Twin mothers also have a higher probability of dying from lung cancer/COPD and heart attacks/strokes. The effects of twins on maternal health are the strongest for high-educated mothers and mothers with above-median pension income.

Our results fit into a recent line of epidemiological and sociological literature on the adverse effects of the work-family strain, which offers three general theoretical considerations. First, there are selection effects: women who are employed, married, and have children are healthier than their childless, unmarried, and unemployed counterparts. Second, according to the role accumulation theory, combining family and work is beneficial for women’s health. Third, multiple role theory states that combining work and family roles leads to stress, with negative consequences for health (Kahn et al. 1964).

In our study, we use twins at first birth as a shock to fertility, which allows us to partly overcome the selection problems arising in this literature. We find that all-cause mortality and mortality from lung cancer and heart diseases are significantly elevated among mothers who give birth to twins. The higher probability of death due to lung cancer and heart diseases indicates that at least part of the effect is stress-related, given that the medical literature strongly associates these causes of death with stress from work-family conflicts and caregiving. That is, the additional burden on women resulting from having two versus one child at their first birth takes its toll on their health later in life.

We also find strong mortality effects among mothers with higher education and above-median pension income. We argue that this can be taken as evidence of a double-burden effect. Highly educated mothers have a higher likelihood of pursuing a career despite having children, and higher pension income indicates that women worked more. In other words, higher education is an *ex ante* predictor of higher labor market attachment, and pension income is a proxy for *ex post* realized labor market activity. Thus, higher all-cause and stress-related mortality rates among mothers with higher education and above-median pension income combine to point in the direction of a double burden from child-rearing and working that negatively affects maternal health in old age.

The specific mechanisms behind these findings deserve further research, especially because women of younger generations are increasingly more likely to stay attached to the labor force while raising children (Goldin and Mitchell 2017). Fathers’ roles in supporting their families, both financially and by taking a more active role in raising children, change as well. Changes in family planning have to be taken into account when it comes to the external validity of our results for younger generations. We shed some light on this issue by depicting the averages of age at first birth, number of children, and birth spacing for younger and older generations in Sweden. In any case, more research is necessary to find policies that can protect mothers from the double burden of family and work. The recent study by Avendano et al. (2015), for example, showed that more generous parental-leave policies reduce maternal depression in old age. This is particularly relevant in times of demographic change, when many countries aim to increase both female labor supply and birth rates.

## Electronic supplementary material

ESM 1 (PDF 1178 kb)

## Footnotes

1 A potential threat to our empirical strategy is that twin births are not random. The probability of a twin birth increases with mothers’ age at birth and the use of in vitro fertilization. We argue that we nevertheless can use this strategy because the cohorts examined in this study had their first children well before any major impact of fertility treatments on the number of twin births. Additionally, we condition on mothers’ age at first birth in all our analyses.

2 For these two strategies, we do not assume that twin births have no effect on mothers’ labor market decisions. For instance, twin mothers may reduce labor market participation, which could have a positive effect on their health. This is one reason why we use these strategies in addition to the initial twin effect as evidence of a double burden.

3 We focus on mothers because women are traditionally more likely than men to experience conflict in family and work life. Additionally, because of the structure of the data set, we can link children to their biological father and mother, but we do not know whether parents are separated. However, we can safely assume that children of separated parents live with their mother most of the time.

4 Ridker et al. (2000) showed that among markers of inflammation, C-reactive protein (CRP) and interleukin-6 (IL-6) are strong predictors of cardiovascular diseases in older women. At the same time, CRP and IL-6 are known to be elevated by chronic stress, such as that experienced from caregiving (Kiecolt-Glaser et al. 2003; Robles et al. 2005).

5 Another alternative mechanism is that higher education may induce more specialized jobs, so that higher educated women may move farther from their families and have different networks. We cannot rule out that this contributes to the more negative health effects for twin mothers with higher education.

6 The overall twin rate (i.e., twins at any birth) is 2.1%. We exclude 14 mothers who gave birth to triplets or more.

7 The ICD-9 is applied in the years 1979–1998, and the ICD-10 from 1999 onward. The codes are lung cancer (ICD-9: 162.2–162.5, 162.8–162.9; and ICD-10: C34), heart attack (ICD-9: 410 and ICD-10: I21), COPD (ICD-9: 490–496 and ICD-10: J40–J43, J44.0–J44.7, J44.9, J45–J48), and stroke (ICD-9: 430–439 and ICD-10: I60–I69).

8 We use the earnings-related part of the pension income (*tilläggspension*) and do not include the basic pension (*folkpension*) in our pension income measure. Pension income is adjusted for inflation.

9 We use pension income at age 72 as a proxy for lifetime income because of data restrictions and selection issues. Specifically, pension income is available only for 2001–2008 and for individuals aged 65–74. Consequentially, for example, if we use pension income at age 65, we can observe pension income for only one of our cohorts. However, if we use pension income at higher ages, we observe pension income for only the survivors, which creates a selection problem if there are mortality differences between twin and nontwin mothers. To maximize the sample size and avoid selection, we use pension income at age 72, which we can observe for seven cohorts (aged 55–61) in 1990.

10 Note that 100,000 SEK corresponds to roughly 10,800 EUR in 2002.

11 The levels of education that we include in the regression model are finer than the three strata we use to condition our sample: 1 = only elementary schooling, 2 = junior secondary schooling, 3 = upper secondary schooling of at most two years, 4 = longer upper secondary schooling, 5 = tertiary education of less than three years, 6 = tertiary education of three years or more, and 7 = PhD.

12 Farbmacher et al. (2018) demonstrated that the probability of giving birth to twins follows an inverted U-shape across age at birth.

13 We still checked for sample selection bias by comparing twin births and schooling outcomes. To this end, we estimated an ordered logit model for level of education (with seven education levels) using twins at first birth, cohort fixed effects, and a quadratic polynomial in age at first birth as explanatory variables. The results, shown in column 3 of Table A1 in the online appendix, suggest that twin births are not a significant predictor of mothers’ level of education.

14 We find similar patterns when we consider twin births at any birth (not shown).

15 We tested the statistical significance of the differences in the effects between educational groups. We found a significant difference (at the 5% level) in the absolute effect of twinning between lower- and higher-educated mothers for overall mortality but not for the stress-related diseases. We found significant differences in the effect of twinning between middle- and higher-educated mothers for all outcomes.

16 As a robustness check, we ran the same analysis for the pension income at age 71 for the cohorts age 55 to 60 in 1990; results were very similar.

17 The difference in effect sizes between mothers with above- and below-median pension incomes is highly significant for all-cause mortality.

18 We use labor income in 1985 because this is the earliest year for which we observe income. Mothers with zero labor income in this year are assigned zero log labor income.
