# Supplementary material for: Double Trouble: The Burden of Child-rearing and Working on Maternal Mortality
Source: Demography. 2020 Apr 8;57(2):559–76. doi: 10.1007/s13524-020-00868-6 (PMC7162835; doi:10.1007/s13524-020-00868-6)
Supplement: Supplementary file 1 — (PDF 1178 kb) [file 13524_2020_868_MOESM1_ESM.pdf]

Double trouble: The burden of child rearing and working on maternal mortality

Online Appendix

Tabea Bucher-Koenen, Helmut Farbmacher
Raphael Guber, Johan Vikström

September 6, 2019

Outcome	(1) Resident in 1990	(2) Died betw. 1961–1990	(3) Education (ordered logit)	(4) Pension at age 72 missing	(5) Pension at age 72 in 100 SEK
twins	0.005	-0.004	-0.014	0.006	-0.716
	(0.005)	(0.004)	(0.037)	(0.005)	(9.627)
same-sex twins	0.005	-0.005	0.046	0.006	-10.162
	(0.006)	(0.005)	(0.046)	(0.006)	(11.632)
Unconditional mean	0.914	0.065	2.224	0.446	817.846
Observations	444,197	444,197	404,286	404,286	209,325

In columns 1,2,4 and 5, each coefficient-standard error pair comes from a single regression of a linear probability model controlling for education, cohort dummies and a quadratic polynomial in age at first birth. Column 3 uses education in seven levels as outcome, is estimated using an ordered logit model and controls for cohort dummies and a quadratic polynomial in age at first birth. Robust standard errors in parentheses below. ***,**,* indicate significance at the 1%, 5%, and 10% level, respectively.

Table A1: Selection into different samples

	(1) All twins	(2) Same-sex twins
# children 5 years after first birth	0.68***	0.69***
	(0.012)	(0.015)
# children 10 years after first birth	0.61***	0.61***
	(0.017)	(0.021)
# children 15 years after first birth	0.59***	0.59***
	(0.019)	(0.023)
Total # children	0.59***	0.59***
	(0.020)	(0.025)

Note: Table displays linear probability models controlling for education, cohort dummies and a quadratic polynomial in age at first birth. Robust standard errors in parentheses below. ***,**,* indicate significance at the 1%, 5%, and 10% level, respectively.

Table A2: Twin births and the number of children

	(1) Twin parents	(2) Non-twin parents
Spacing between first and second child	0	4.00
Spacing between first and last child	3.80	7.38
Spacing between second and last child	3.80	3.37

Note: Twin parents defined by twinning at first birth. Sample statistics for the sample as described in Section 2.

Table A3: Twin births and child spacing (averages in years)

	(1) Died between 1991 and 2010	(2) Lung cancer/ COPD	(3) Heart attack/ stroke	(4) Died between 1991 and 2010	(5) Lung cancer/ COPD	(6) Heart attack/ stroke
**Panel A: All twins**						
Twins	0.027**	0.018***	0.021**	0.027**	0.018***	0.020**
	(0.012)	(0.006)	(0.010)	(0.012)	(0.006)	(0.010)
Twins	0.004	-0.021**	-0.021	0.006	-0.021**	-0.019
× upper secondary schooling	(0.020)	(0.009)	(0.015)	(0.020)	(0.009)	(0.015)
Twins	0.057**	0.004	0.020	0.054**	0.004	0.021
× tertiary schooling	(0.026)	(0.012)	(0.019)	(0.026)	(0.012)	(0.019)
**Panel B: Same-sex twins**						
Same-sex twins	0.027*	0.019**	0.023*	0.027*	0.020**	0.022*
	(0.015)	(0.008)	(0.012)	(0.015)	(0.008)	(0.012)
Same-sex twins	0.000	-0.024**	-0.026	0.024	-0.024**	-0.024
× upper secondary schooling	(0.024)	(0.011)	(0.018)	(0.024)	(0.011)	(0.018)
Same-sex twins	0.072**	0.001	0.032	0.067**	0.000	0.032
× tertiary schooling	(0.032)	(0.015)	(0.024)	(0.032)	(0.015)	(0.024)
Control for income	no	no	no	yes	yes	yes
Unconditional mean	0.287	0.044	0.136	0.287	0.044	0.136
Observations	404,286	404,286	404,286	404,286	404,286	404,286

Note: Table displays linear probability models controlling for finer levels of education, cohort dummies and a quadratic polynomial in age at first birth. Columns 4 to 6 additionally control for a quadratic polynomial in log-labor market income in 1985. Individuals with zero income in 1985 are assigned a value of zero. The reference group are mothers with primary education. Robust standard errors in parentheses below. ***,**,* indicate significance at the 1%, 5%, and 10% level, respectively.

Table A4: Interaction model for education

	(1) Died between 1991 and 2010	(2) Lung cancer/ COPD	(3) Heart attack/ stroke
**Panel A: All twins**			
Twins	0.010	0.002	0.005
	(0.012)	(0.005)	(0.010)
Twins × above median pension income	0.037*	0.015*	0.015
	(0.009)	(0.009)	(0.014)
**Panel B: Same-sex twins**			
Same-sex twins	0.019	0.006	0.004
	(0.016)	(0.007)	(0.011)
Same-sex twins × above median pension income	0.031	0.009	0.034*
	(0.024)	(0.012)	(0.018)
Unconditional mean	0.287	0.044	0.136
Observations	404,286	404,286	404,286

Note: Table displays linear probability models controlling for finer levels of education, cohort dummies and a quadratic polynomial in age at first birth. The reference group are mothers with primary education. Robust standard errors in parentheses below. ***,**,* indicate significance at the 1%, 5%, and 10% level, respectively.

Table A5: Interaction model for pension income

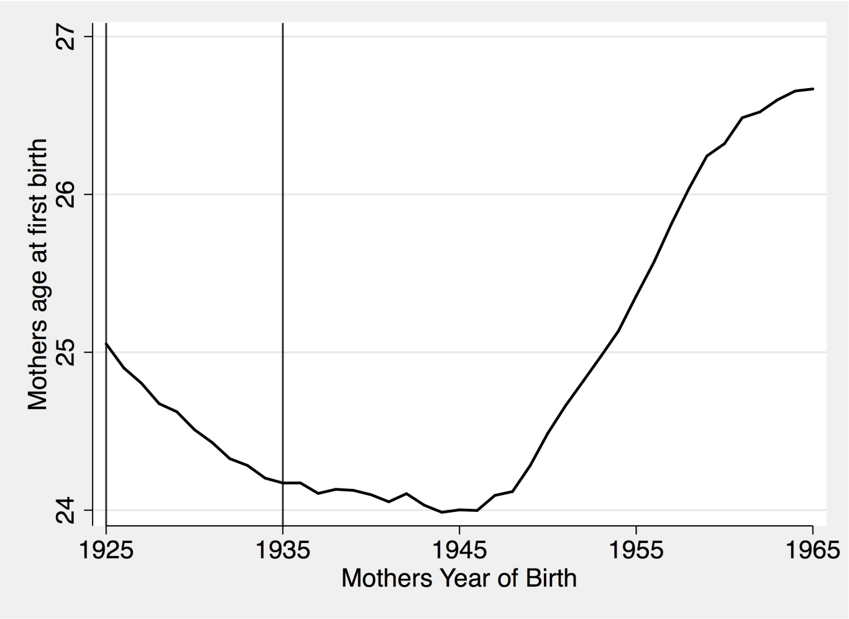

Figure A1: Average age of a mother at first birth

Note: The vertical lines indicate the mothers in our sample.

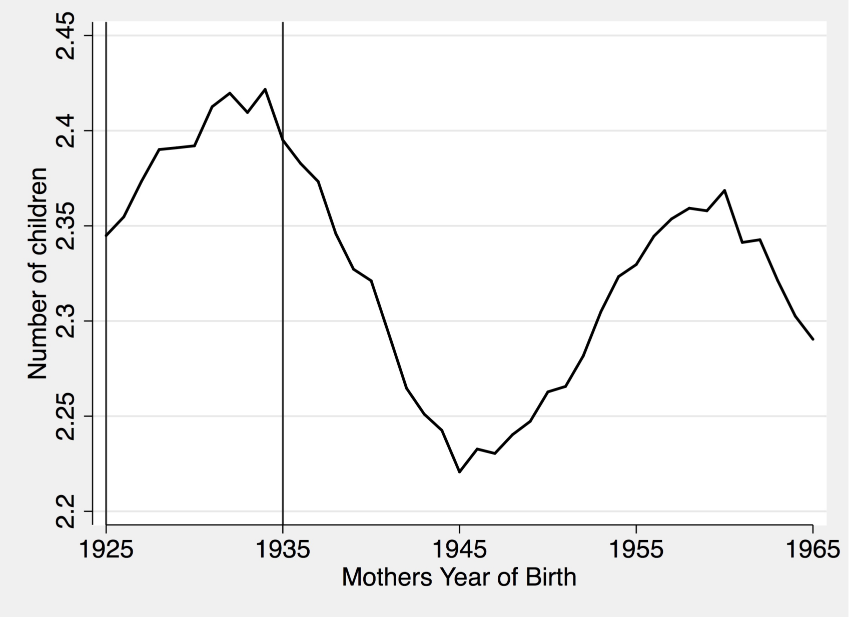

Figure A2: Number of children per woman

Note: The vertical lines indicate the mothers in our sample.

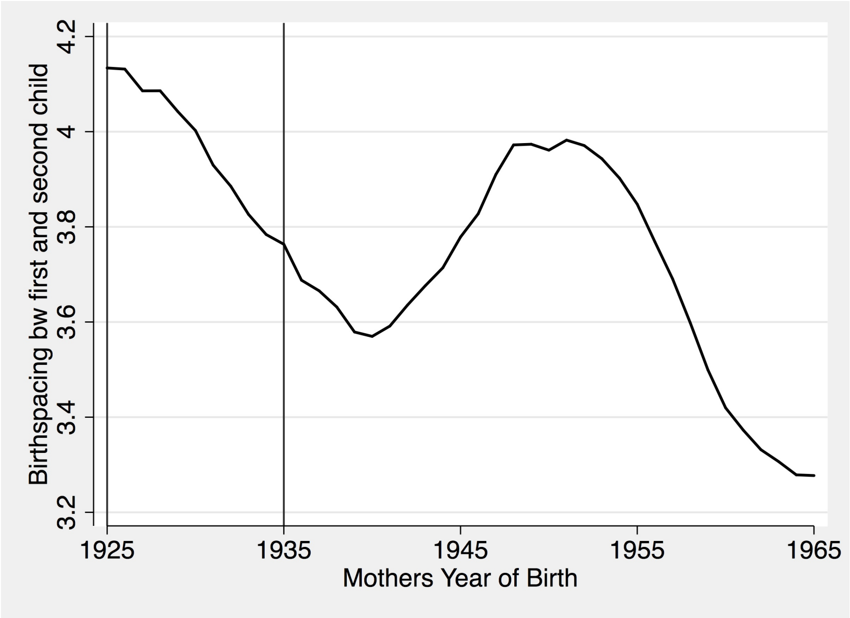

Figure A3: Birth spacing (in years) between first and second child

Note: The vertical lines indicate the mothers in our sample.

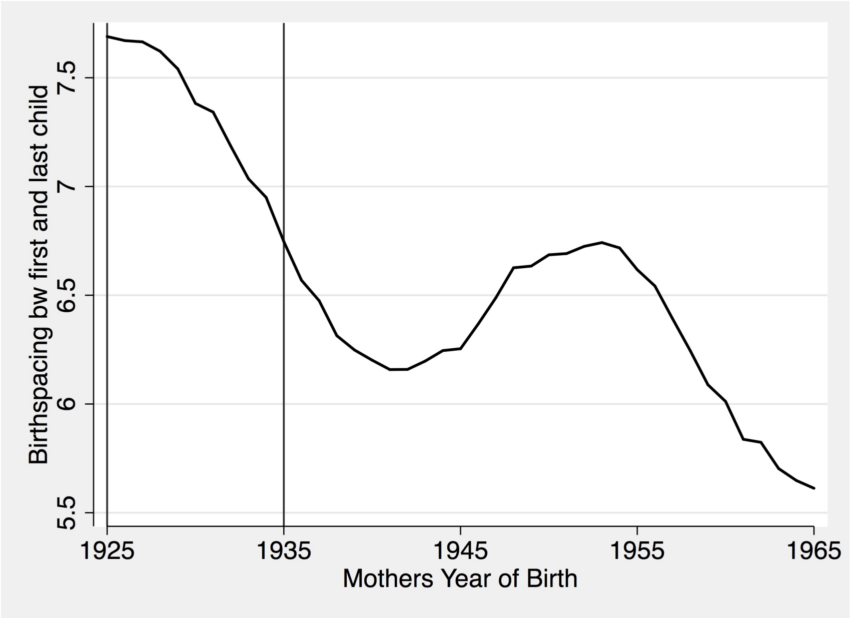

Figure A4: Birth spacing (in years) between first and last child

Note: The vertical lines indicate the mothers in our sample.
